# A multilevel carbon and water footprint dataset of food commodities

**DOI:** 10.1038/s41597-021-00909-8

**Published:** 2021-05-07

**Authors:** Tashina Petersson, Luca Secondi, Andrea Magnani, Marta Antonelli, Katarzyna Dembska, Riccardo Valentini, Alessandra Varotto, Simona Castaldi

**Affiliations:** 1 Università Degli Studi Della Tuscia, DIBAF, via Camillo de Lellis 4, 01100 Viterbo, Italy; 2 Barilla Center for Food & Nutrition Foundation, via Madre Teresa di Calcutta, 3/a, Parma, Italy; 3 Euro-Mediterranean Centre on Climate Change, via Augusto Imperatore 16, 73100 Lecce, Italy; 4 Università Degli Studi Della Campania “Luigi Vanvitelli”, DISTABIF, via Vivaldi 43, 81100 Caserta, Italy

**Keywords:** Environmental impact, Climate-change mitigation, Agroecology

## Abstract

Informing and engaging citizens to adopt sustainable diets is a key strategy for reducing global environmental impacts of the agricultural and food sectors. In this respect, the first requisite to support citizens and actors of the food sector is to provide them a publicly available, reliable and ready to use synthesis of environmental pressures associated to food commodities. Here we introduce the SU-EATABLE LIFE database, a multilevel database of carbon (CF) and water (WF) footprint values of food commodities, based on a standardized methodology to extract information and assign optimal footprint values and uncertainties to food items, starting from peer-reviewed articles and grey literature. The database and its innovative methodological framework for uncertainty treatment and data quality assurance provides a solid basis for evaluating the impact of dietary shifts on global environmental policies, including climate mitigation through greenhouse gas emission reductions. The database ensures repeatability and further expansion, providing a reliable science-based tool for managers and researcher in the food sector.

## Abstract (summary)

**Table:** 

Measurement(s)	carbon footprint of food products and related uncertainties • water footprint of food products and related uncertainties
Technology Type(s)	digital curation • statistical analysis
Factor Type(s)	Typologies, sub-tyologies and items of food products
Sample Characteristic - Environment	agroecosystem • food production system
Sample Characteristic - Location	global

Machine-accessible metadata file describing the reported data: 10.6084/m9.figshare.14115608

## Background & Summary

Food is essential for human physical and psychological wellbeing, it is a driver of economy and a key element of cultural identity and human heritage. Food also plays a relevant role in present and future world sustainable development strategies, as the food chain accounts up to 37% of the global greenhouse emissions^1^, 70% of water withdrawals^2^ and strongly contributes to deforestation, mineral depletion, desertification, eutrophication, acidification, biodiversity loss and genetic erosion^3,4^. Each food product brings its share of generated environmental pressures during food production, processing, distribution and end-life^5^. The degree of such environmental burden varies significantly with food type and with food production characteristics^6^. However, only few indicators of food pressure have standardized procedures, the two most well-known being the carbon footprint^7^ (CF) and the water footprint^8^ (WF). The CF of a food product represents the total greenhouse gases emitted during all the phases of its production and distribution quantified using a life cycle approach. It is a straightforward indicator of the share of global GHG emissions that can be attributed to food producers or consumers and can be easily linked to climate policies. The WF of a food product is the amount of water that is consumed and polluted in all processing stages of its production. It is a consolidated indicator, frequently used to compare the relative impacts of different food types and dietary habits^9^.

A global transition towards sustainable food production and consumption is among the most beneficial global strategies proposed to mitigate human impact on planet resources^1,3^ and it is a fundamental asset of the new EU strategy “Farm to Fork” as part of the EU Green Deal strategy. A clear link has been demonstrated between healthy and sustainable dietary choices^3^, providing a win-win opportunity for all the stakeholders of the food sector, from consumers to food providers. The first step to support societal engagement on sustainable diets is to provide knowledge and tools that can allow turning ideas into action. Several studies and analytical reviews of food CF and WF footprints have provided information on different dietary impacts^3,6,10^, methodological issues^6,11,12^, policy recommendation^6,13^. A further step is to offer an open access database aimed at different kinds of stakeholders, from scientific experts to business companies, citizens and policymakers, to be used for calculations, management and reporting. To meet this goal, we created a multilevel database of CF and WF of food items, based on a standardized methodology to assign scientifically meaningful values of footprint to food commodities, starting from publications reported in literature. Structure and methodological approach, used to create the database, easily allow for further expansion or reorganization of specific food items and typologies depending on the specific user’s need. As such it provides a flexible science-based tool for operators, managers and researcher in the food sector.

**Fig. 1:**
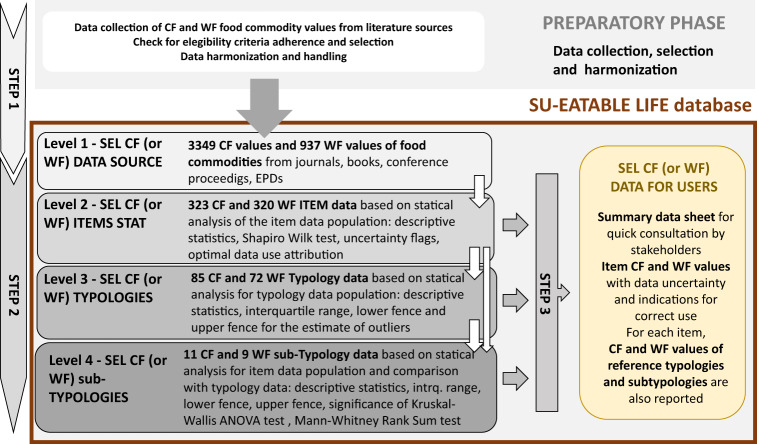
General outline of the different construction steps of SU-EATABLE LIFE (SEL) database. Step 1 includes the preparatory phase where studies were collected from literature and public repositories, selected on the basis of eligibility criteria, CF and WF values of food commodities extracted and harmonized, and then reported into the level 1 information of the SEL database. In Step 2 the other layers of information are created which represent different levels of aggregation of data reported in level 1. CF and WF values statistical analysis are reported for food items (level 2), typologies (level 3) and sub-typologies (level 4). In Step 3 the complex set of data reported in Level, 1, 2 and 3 are summarized into an easy to use dataset suitable for quick consultation by technical and not technical users.

The database, developed in the framework of the EU SU-EATABLE LIFE project, builds on the double pyramid database^14^ of the Barilla Center for Food and Nutrition. The SU-EATABLE LIFE (SEL) database is organized in different levels of information (Fig. 1) suitable for different users and purposes. Level 1 reports CF and WF data of food commodities extracted from published studies and harmonized to refer to the same system boundaries and functional units. Each data is conceptually associated to a food item name, to a food typology, sub-typology and to a food group (Table 1).

**Table 1:** Hierarchical classification applied to CF and WF data in the SEL database.

Level of aggregation	Description of aggregated food commodities
**Group**	Wide category of food commodity. The database includes 4 groups. 1) *Agricultural processed* covering manly plant based industrial processed food. 2) *Animal husbandry* covering products of terrestrial animal origin. 3) *Crops* covering fresh or minimally processed plant based products like dried, canned, frozen vegetables and fruit. 4) *Fishing* which includes products from fresh and salted waters.
**Typology**	Aggregated level of food commodity as generally known in the food system. It represents a group of items having similar characteristics. For example the typology “legumes” includes peas, lentils, beans, soybeans etc.
**Sub-typology**	Used to subdivide three typologies (vegetables, fruit, shellfish) which include a large variability of items (i.e. the typology “shellfish” includes the sub-typologies crustacean, bivalves and cephalopods).
**Item**	It is the most detailed level for aggregated data. Generally, the item name corresponds to common market definitions (ex. tomato, mussels, milk). The level of detail proposed depends on the available data source.

This hierarchical assignment allows shifting easily among different levels of aggregation of food data information. At level 2, data from single studies of level 1 are aggregated to represent food “item” values and associated uncertainty, which support the user in determining how robust are the available data for technical and scientific purposes. Level 3 is a further level of data aggregation, where item values from level 2 are aggregated based on their “typology” identifier. Level 4 is another possibility to represent a food commodity using the value of sub-typologies, which are created when a food typology is quite broad. A distillation of all the information contained in the level 2, 3 and 4 is provided into an easy to use data sheet, SEL CF or WF DATA FOR USERS (Fig. 1).

## Methods

With the aim of obtaining a useful tool for stakeholders to explore, assess and use the information related to CF and WF of food commodities, we implemented a multi-step methodological framework to create an easy to use CF and WF repository of food items, which can be expanded or modified for tailored requirements using a science based approach for each step of its creation (Fig. 1).

The overall methodological procedure is made of 3 steps. *Step 1* is related to CF and WF data collection from literature, eligibility check and harmonization, to create the base level of the database (level 1). *Step 2* is about the creation of other three informative layers with higher level of data aggregation. These might be the data of direct interests for stakeholders of the food systems. A rigorous statistical approach is proposed to evaluate the quality of analysed data and criteria for the correct use of data, based on statistical evidence, are set and applied to the data. In *Step 3* the complex set of statistical evaluations, done for each informative level, is summarized into an easy to use dataset reporting values of CF and WF of food items. Thanks to its multilevel approach, the database provides a flexible tool for different purposes and levels of expertise. Each step is based on transparent procedures that allow users to replicate, to implement and to modify each level of the database.

The three steps are described in details in the following paragraphs.

### Step 1 - CF and WF data collection, harmonization and compilation of level 1 of SEL database

The first step was to review the published data of CF and WF of food commodities. We revised literature data published till January 2020 including peer-reviewed papers, conference proceedings, public reports or studies where methods of data collection and handling were described, and Environmental Product Declarations (EPDs).

For the collection of CF data, a significant input came from the systematic review of Clune *et al*.^11^, who reviewed 369 published studies, covering the period 2000–2015, proving 168 varieties of fresh food products based on 1718 data entries. An additional source of studies reporting both CF and WF was the Double Pyramid database 2016 built on the previous version 2014^14^ (BCFN2016 https://www.barillacfn.com/en/publications/double-pyramid-2016/), which reports 1202 CF values from 468 sources covering 240 food items and 309 WF values from 136 data sources covering 152 food items (reference period 1998–2016). Part of CF data of this latter dataset, up to year 2014, were already revised and included in the Clune *et al*.^11^ study. To avoid double counting from these two sources, data from both sources were checked for authorship, plus the CF reported data were compared and if in disagreement the original data were checked in the paper. Data reported in the Double Pyramid database 2016 but not present in Clune *et al*.^11^, mostly referring to processed food, were checked for eligibility applying the exclusion criteria reported in Table 2 and if considered eligible they were included in the present database.

**Table 2:** Exclusion criteria to be applied to CF and WF data collected from literature to create SEL database level 1.

Exclusion criteria list
• Studies not reported in public databases.
• Internal not published calculations.
• Studies which do not properly specify objectives, methods, and results (Prisma protocol^30^).
• CF studies not based on life cycle methodological approach.
• Studies which calculate WF as ‘consumed water’, ‘water use’ or simple ‘blue water’ and are not based on the methodology to estimate the water footprint as reported by Hoekstra *et al*.^31^
• CF studies where the functional unit is not expressed as kg CO_2_ equ kg^−1^ or litre of product or where it is not possible to derive the functional unit.
• WF studies where the functional unit is not expressed as litres of water/kg or litre of product or where it is not possible to derive the functional unit.
• CF studies with system boundary beyond the distribution centre of the final product and which do not report the contribution of different life cycle stages to the total CF.

A new literature search was done to integrate data not covered by the previous reviews using three online bibliographic sources SCOPUS (https://www.scopus.com/home.uri), Google Scholar (https://scholar.google.com/) and the Google search engine (https://www.google.com/), which was concluded in January 2020. To search the bibliographic sources, we used the combinations of two sets of words. The first set referred to “impacts” and included the following words: *carbon footprint*, *water footprint*, *virtual water*, *greenhouse gases*, *environmental impact*, *life cycle*, *LCA*, *LCI*, *EPD*. The second set referred to “products” and included words like *food*, *beverages*, *fish*, *shellfish*, *crops*, *vegetables*, *fruit*, *meat*, *eggs*, *dairy*. EPDs were updated based on data reported on the International EPD’s System database (www.environdec.com). Added studies were evaluated for exclusion criteria (Table 2).

The final list of data from single studies reported in the SEL database was distributed as follow: 3349 CF data, including 1397 data of fresh food commodities already reported in Clune *et al*.^11^, 803 CF data originally reported in Double Pyramid 2016 database, which were checked for eligibility and harmonized, and 701 CF data added with this study; 938 WF data, including 288 WF data originally reported in double pyramid 2016 and 650 WF data added with this study.

All the CF and WF values extracted from the collected studies were assigned a group, a typology, a sub-typology when this applied, and an item name (Table 1) and were recorded on an excel sheet including the following additional information: type of bibliographic source, full reference, publication year, system boundary at distribution, country of production, region of production, relevant notes, presence of the same value in other data collections (i.e. Clune *et al*.^11^ or Double Pyramid 2016).

After data collection, CF data where further analysed and handled for the harmonization of the system boundary following the approach as reported in Clune *et al*.^11^. The system boundary considered in the SEL database is the distribution centre to consumers located in the country of origin. It hence excludes post market phase like for example cooking. The system boundaries at distribution have a wide range of specifications in the published papers. We accepted regional distribution centre (RDC), international distribution centre (IDC), European distribution centre (EDC), country ports of final destination, warehouses, wholesalers, city markets, up to retailers. For the specific case of international transport, which includes also the emissions for shipping to regional distribution centres of the hosting country, rather than excluding the studies we have created a dedicated typology “imported”, which however includes very few studies. The imported commodity is indicated in the SEL database by a capital letter “I”.

If CF values collected from literature referred to the system boundary “farm gate” or “slaughterhouse”, additional post farm gate GHG emissions were added as proposed by Clune *et al*.^11^. These additional emissions also included packaging if not reported in the publication. We adopted the median value for distribution to RDC (0,09 kg CO_2_/kg or kg CO_2_/L) and packaging (0,05 kg CO_2_/kg or kg CO_2_/L) used by Clune *et al*.^11^. Data referring to slaughterhouse emissions were also taken from the same publication.

To address the share of WF for packaging and transportation to the market we analysed 256 EPD’s. No significant increase of WF in downstream stages associated to packaging and distribution was found. Thus we included in the analysis all system boundaries with the exception of ‘cooking’, human excretion and waste disposal.

To transform CF values from carcass or live weight to bone free meat, ratios reported in in Clune *et al*.^11^ were used, while the ratio carcass weight to bone free meat for buffalo meat (1:0.684) was estimated from the studies of Gerber *et al*.^15^, Gurunathan *et al*.^16^, Li *et al*.^17^.

The final version of CF and WF data, after data handling was recorded in a sheet where, in addition to the information mentioned above for each study, we also reported additional post farm gate emissions (transport T, slaughtering S, packaging P) or meat conversion factors (cf) when applying. This complete dataset represents the *level 1* information sheet of the SEL database (Fig. 1).

A change in 100-year global warming potential (GWP) factors provided by the International Panel on Climate Change reports AR3 (2001), AR4 (2007) and AR5 (2013) might have introduced additional variability in the studies of LCA on which CF data of level 1 are based. The extent of such variability is difficult to quantify as it depends on the relative weight of each GHG on the total CF of the item. However, the analysis of some item groups (tomato, rice, beef meat, chicken meat), used as sample test, did not show any clear trend of CF average reduction or increase over the years (1998–2020), suggesting that differences among production processes and conditions were the dominant source of CF variability.

### Step 2 - Creation of derived CF and WF datasets with higher aggregation level (2, 3 and 4)

This step provides footprints of food commodities with a higher level of aggregation corresponding to food items, typologies and sub-typologies (Table 1), which might be of particular interest for different kinds of stakeholders. The item represents the higher detail of aggregated footprint data of a food commodity and it is often the most desirable information for food impact analysis and dietary assessments. We propose here a methodological framework to evaluate the uncertainty associated to data used to represent food items. The methodological framework will support the users in their choice of the optimal value to represent the food item on the basis of the available data present in the database. It also would easily allow for expansion and implementation of food item values.

#### Level 2, SEL CF ITEM & SEL WF ITEM datasets

These two datasets (CF and WF) report a comprehensive set of descriptive statistics for the list of food items present in the database. The population of data used to attribute a value and uncertainty to a food item is made of all the CF or WF values classified with that “item entry name” in the dataset of *level 1* of SEL database.

The item data population is described in *level 2* by the following set of information.

**Size:** number of studies used for the analysis of item population (n).

**Location and central-tendency measures:** in terms of mean, median, first quartile (Q1) and third quartile (Q3), including also the minimum (Min) and maximum (Max) observed values.

**Variability measures:** Standard Deviation (SD) Coefficient of Variation (CV) as absolute and relative dispersion indexes, the Interquartile Range (IQR) and the Median Absolute Deviation (MAD) as more robust indexes of variability.

**Shape measures:** Skewness (SK), kurtosis (KU) indexes and Shapiro-Wilk normality test (SW test).

The **median** of the item data population was chosen to assign a value of central tendency which represents the item. The median offers the advantage of not being influenced by the presence of outliers which misrepresent the value of the mean, making it a less meaningful measure. As such, the median represents the location estimator with the highest breakdown point (equal to 0.5) and with “the maximum proportion of observations that can be contaminated (i.e., set to infinity) without forcing the estimator to result in a “false” and not-representative value^18,19^. With these properties, the median also represents the most appropriate measure of central tendency to describe both positively and negatively skewed distributions^20^.

To describe the uncertainty associated to the position value (median) we used descriptive statistic data relative to dispersion and shape of item data distribution. In particular, we used skewness and kurtosis indexes, which gave us information on the existence of symmetric or skewed distributions, as well as on their ‘peakedness’ measured as relative to the weights of the tails^21^, thus enabling us to evaluate (for each distribution) the importance of extreme values over the entire set of data and the related level of dispersion (platykurtic versus leptokurtic distributions). We completed the shape analysis by carrying out the Shapiro-Wilk test^22,23^ (4 ≤ n ≤ 2000).

**Fig. 2:**
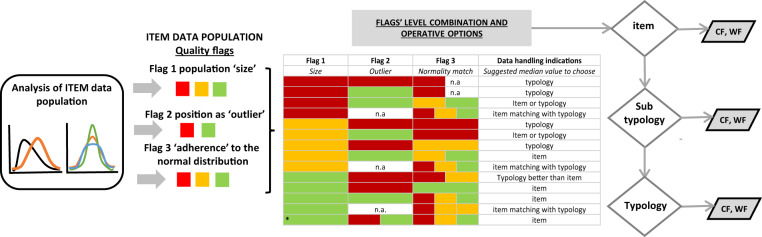
Method for attribution of CF (or WF) value to a food item based on data quality flags. The scheme shows the procedure applied to evaluate the level of uncertainty associated to CF or WF value of a food item and how this information is used to decide the best value that should be used to represent the item. Three quality flags related to a statistical aspect of the data population are calculated to attribute the level of uncertainty. Each flag has different level of quality, red being the worst, green the best. Flags are then combined and expert judgement is used to associate a suggestion for data use to each flag combination. If the item median value is characterized by high uncertainty it poorly represents the item and caution is needed to use this data to represent the food commodity, the users is therefore redirected to a higher level of aggregation such as the sub-typology or the typology which includes the analysed item.

To define the uncertainty of the item value we created an assignment method based on a combination of the three quality flags (Fig. 2).

#### Flag 1, evaluation of the ‘size’ (n) of the “item data population”

- ***Red if*** **n < 4**, as three is the minimum number of observations needed for distinguishing the median from the mean and for evaluating the approximation of the empirical distributions to known parametric distributions, in accordance with the minimum requirements specified by Royston^23^.
- ***Yellow if*** **4 < n ≤ 8**, minimum level of observations needed for jointly evaluating kurtosis and skewness of a distribution^24^.
- ***Green if*** **n > 8** number of observations required to identify cases in which location (as well as variability and shapes) measures can be properly computed and evaluated.
- Green with asterisk (*): applies to WF values which are global or regional or national average estimates and green is assigned independently by “n”. In this case the flags 2 and 3 do not apply for the evaluation of the WF item value.

#### Flag 2, evaluation of outlier position of items characterized by RED or YELLOW Flag 1

This flag is used to test if items with population size n ≤8, are outliers for their respective typology population. If they are outliers they cannot be alternatively represented by the typology value, as they will be particularly higher or lower than the item data population representing the reference typology. An example of this in the SEL database is the CF value of the item “lobster” which has a yellow flag 1 (n = 5). The item lobster is an outlier for the typology “shellfish”. In this case even if there is a certain level of uncertainty in the “lobster” item value, it is not advisable to substitute this value with the typology value “shellfish”.

To attribute flag 2 output, the Tukey’s rule^25^ was used. The outlier identification is based on the quartiles of data distribution, where the first quartile Q1 is the value ≥1/4 of the data, the second quartile Q2 or the median is the value ≥1/2 of the data, and the third quartile Q3 is the value ≥3/4 of the data. The interquartile range, IQR, is Q3 − Q1. The data used to estimate quartile values are the medians of the items composing the typology population.

- **Red** if the median value of the analysed item (x), is an outlier, i.e. following the Tukey’s rule it is more than 1.5 times the interquartile range from the quartiles, either x < Q1 − 1.5 IQR, or x > Q3 + 1.5 IQR.
- **Green flag** if the median value of the uncertain item analysed (x), is not an outlier, i.e. it is within 1.5 times the interquartile range from the quartiles, Q1 - 1.5 IQR < x < Q3 + 1.5 IQR.
- **NA**, **no flag**: the Tukey rule was not applied because the items coincide with the typology, i.e. the typology is only made by this sole item for the time being.

#### Flag 3 adherence to the normal distribution

It evaluates the level of dispersion and clustering of the observed data points to the centre. To test the adherence of the item data population distribution to the normal distribution, the Shapiro-Wilk test was carried out. The three following colours were assigned:

- **Red:** characterizing those items: i) whose size was lower than 4, thus preventing the evaluation of normal distribution approximation, as detailed above; ii) items for which we rejected the null hypothesis of bell-shaped distribution at the 1% level of significance (p-value < 0.01), therefore highlighting substantial asymmetric distribution and/or a heavy-tail distributions characterized by a level of clustering (low or high) not adequate to describe the findings with a synthetic measure, computed at the same item level.
- **Yellow:** for those items whose empirical distribution, even if departing from the Normal distribution, lead us to reject the null hypothesis with a greater level of errors (0.01 ≤ p-value < 0.05).
- **Green**: for those items whose empirical distribution lead us to not reject the null hypothesis, therefore confirming the validity of central tendency measures (at the item level) to be used for summary description.
- **NA no flag:** the Shapiro-Wilk test could not be run due to an insufficient number of data in the population (n < 3).

The outputs of the three flags were considered together to evaluate the uncertainty related to the median CF or WF value of food items, and based on the level of uncertainty indications for an optimal data use were provided (Table 3).

**Table 3:** Flag output table.

Flag 1	Flag 2		Flag 3			Data handling indications
*Size*	*Outlier*		*Normality match*			*Suggested median value to represent the food commodity*
**Red**	**Red**		**Red**	n.a		typology
**Red**	Green		**Red**	n.a		typology
**Red**	Green		**Yellow**	**Green**		Item or typology
**Red**	n.a		**Red**	**Yellow**	**Green**	item matching with typology
**Yellow**	**Red**		**Red**			typology
**Yellow**	Green		**Red**			Item or typology
**Yellow**	**Red**		**Yellow**			typology
**Yellow**	**Red**		**Green**			item
**Yellow**	**Green**		**Yellow**	**Green**		item
**Yellow**	n.a		**Red**	**Yellow**	**Green**	item matching with typology
**Green**	**Red**		**Red**	**Yellow**		Typology better than item
**Green**	**Red**		**Green**			item
**Green**	**Green**		**Red**	**Yellow**	**Green**	item
**Green**	n.a.		**Red**	**Yellow**		item matching with typology
Green*	**Red**	**Green**	**Red**	**Yellow**	**Green**	item

Combination of flag colours, attributed to the item data population, into recommendations for the optimal use of the footprint values. Multiple colours in one cell indicate multiple outputs for this cell that can be associated with colours of the other two cells in the same raw; n.a. indicates that the test could not be run (see methods for details), the asterisk * indicates that the WF value of the item is a global or regional or national mean.

A brief rational of the data handling indication is as follow:

1. *item:* the item statistics are sufficiently robust. The user can use the item median value to represent that food commodity.
2. *item or typology:* although the population used to derive the median of the item is reduced in size and its distribution does not optimally fit a normal distribution, the median of the item is not an outlier for the typology population, i.e. the value of the item in not exceptionally high or low compared with other items present in the typology of reference. The user can use either the item median value or the typology median value to assign a footprint value to the chosen item.
3. *item matching with typology:* the typology coincides with one single item, the two objects item and typology, represent hence the same food commodity. The user will find the same median value in the Item and Typology tables, the choice is hence univocal. In this case the level of uncertainty can be estimated from flag 1 and flag 3 because flag 2 cannot be calculated (n.a.).
4. *typology better than item:* different statistical combinations could lead to this option. The uncertainty associated to the item value is sufficiently high to prefer the typology value to represent the food commodity although the item value is not to be discarded.
5. *typology (or sub-typology):* the high uncertainty suggests precaution in using the item value to represent the food commodity and alternatively a higher level of aggregation for this food commodity can be used. When the indication suggests both typology or sub-typology is because there is no statistical difference between the two values and the user can choose which one to use.

#### Level 3, SEL CF Typologies & SEL WF Typologies

In this informative level descriptive statistics is reported for CF and WF data of food typologies. The CF or WF data population of “typologies” is composed by the CF or WF median values of each item that is included in the typology. The value ‘n’ hence represents the number of items in the typology. The same statistical parameters reported for the item relative to size, location and central-tendency measures and variability measures are reported for typologies. The flag approach is not used to test typologies because independently from the uncertainty there is no other meaningful footprint data at higher hierarchical level that can be attributed to a food commodity without losing its specificity. The users can choose to create different typologies from the ones proposed using CF and WF data provided at level 2 (items).

#### Level 4, SEL CF sub-Typologies & SEL WF sub-Typologies

Sub-typologies represent a subgroup of typologies, which have been used when the typology refers to a wide range of food items which could have very different CF and WF values on the basis of some commodity characteristic.

**Fig. 3:**
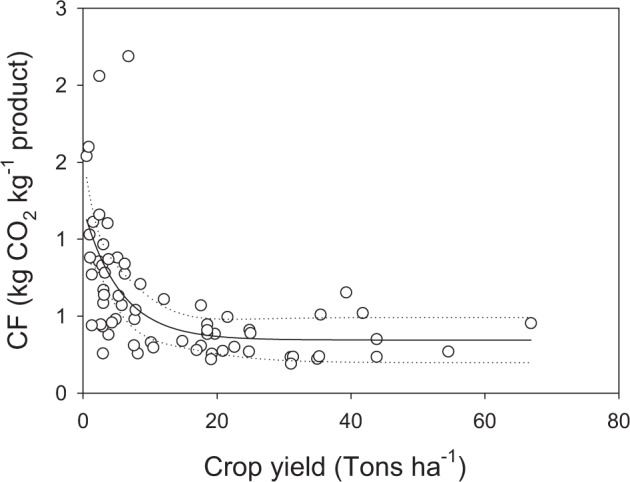
CF value of vegetables vs. their yield. Carbon footprint value of food items included in the typology “vegetables outdoor” is plotted versus their average yield value as reported in FAOSTAT (data EU-28, year 2017).

An example is represented by fresh crop products where the yield per hectare strongly influences the CF value (Fig. 3).

In the SEL database only 3 typologies have been further divided into sub-typologies, as they include very different items in terms of their potential LCA outputs. These are vegetable outdoors, fruit outdoor, and shellfish. The population of data used to evaluate the descriptive statistic for each sub-typology is composed by the median of each item that is included in the sub-typology. Additional statistical information in this informative level 4 are the output of the Kruskal-Wallis ANOVA test on ranks^26^ (all pairwise multiple comparison procedures based on Dunn’s Method^27^) to determine if the median values of the sub-typologies within one typology were significantly different from each other, while the Mann-Whitney Rank Sum Test^28^ was used to determine if a sub-typology footprint value was significantly different from its reference typology data population.

### Step 3 - Creation of summary sheet for easy and quick consultation of CF and WF values of food commodities (SEL CF or WF data for users)

The two summary sheets (CF and WF data) can be considered the most interesting and innovative output of the database, as they translate the complex series of data reported in the 4 informative levels of the database into a list of footprint values of food based on statistical robust analysis. Footprint values of the food items are represented with additional information about value robustness and, where the uncertainty is high, alternative values with higher levels of aggregation are proposed. The summary sheets are meant to provide a scientifically robust and easy to use tool for experts and not experts who want to analyse the impact of food commodities and dietary plans. The users are free to accept the expert-based suggestions or to make their own considerations.

#### Data summary

At present, the SEL database contains 3349 carbon footprint values extrapolated from 841 publications (1998–2019) and 937 water footprint values extrapolated from 88 publications (2005–2018). The CF data are summarized into a total of 85 typologies, 11 sub-typologies, 323 items. WF data are summarized into a total of 72 typologies, 9 sub-typologies, 320 items. A detailed breakdown of the CF and WF into the four food commodity groups, agricultural processed, animal husbandry, crop and fishing is reported in Table 4.

**Table 4:** Number of CF and WF data of food commodities reported in the database informative levels as items (level 2), typologies (level 3) and sub-typologies (level 4).

	Carbon footprint			Water footprint		
	Typol	Sub-typol.	Items	Typol	Sub-typol	Items
**Agri**. **processed**	41	—	100 (570)	33	—	95 (328)
**Animal husb**.	21	—	46 (1530)	22	—	67 (308)
**Crops**	18	8	117 (986)	15	8	136 (246)
**Fishing**	5	3	60 (263)	2	1	22 (55)
***Total***	*85*	*11*	*323* (*3349*)	*72*	9	*320* (*937*)

Their breakdown into the 4 food commodity groups is reported. In brackets are reported the total number of data entries from level 1 used to calculate the food item values.

In terms of geographical distribution, the source data of CF have a Eurocentric prevalence while the WF data are more evenly distributed among America, Asia and Europe, the relative contribution depending on the commodity group (Table 5).

**Table 5:** Geographical distribution of CF and WF data sources as reported in level 1 of the database.

Region	% Geographical distribution of CF and WF data reported in level 1							
	Carbon footprint				Water footprint			
	Agricultural processed	Animal husbandry	Crops	Fishing	Agricultural processed	Animal husbandry	Crops	Fishing
**Africa**	0.5	1.4	—	2.3	0.3	—	2.0	—
**America**	11.9	14.6	17.9	8.0	1.5	21.1	5.7	—
**Asia**	3.7	2.4	11.2	7.6	0.9	16.9	13.4	—
**Europe**	22.4	55.5	40.4	40.4	14.6	16.5	6.1	—
**Mediterranean**	57.7	14.5	19.7	19.7	56.4	15.2	8.5	—
**Oceania**	0.5	10.4	6.4	3.8	—	9.1	1.6	—
**Oceans**	—	—	—	2.8	—	—	—	23.6
**World**	3.1	1.0	—	0.4	26.2	21.1	62.6	76.4
**Not specified**	—	0.1	0.3*	—				

*no EU Countries.

#### Potential applications of the database

The SEL database was created based on the necessity to estimate the CF and WF values of food recipes for meals served in canteens during a set of experiments run in the framework of the EU SU-EATABLE LIFE project aiming at engaging citizens on healthy and sustainable diets to reduce greenhouse gas emissions and water use in EU. During the experiments, researchers and canteen managers were faced with the problem of lacking a quick tool to calculate the food-related environmental impacts based on reliable and science-based estimates. The SEL database was created with this purpose and the summary tables have been used for quick decision making of sustainable recipes and data management. Following this experience, the SEL database would give a significant contribution to bridge the gap between the scientific knowledge provided by the scientific community and the actors of the food system. This historical moment sees a growing attention of the food providers and food consumers to improve the sustainability of food systems. The complexity and challenges of the climate mitigation policies, aiming to reduce greenhouse gas emissions, may find in dietary shift to more sustainable patterns an additional useful element to achieve the policy targets set out by the Paris agreement.

Many data related to the impact of food commodities are available as public or private repositories or publications, ranging from complex scientific studies^3,6,11^ to online anonymous data. The complexity to extrapolate food commodity data from scientific publications and the necessity to evaluate robustness and scientific value of the data available to users requires from the scientific community and extra effort to provide both meaningful data and a scientific methodology for stakeholders from technical, scientific and policy sectors to easily implement and expand the database with a verified and scientifically sound approach. With its different layers of information, the SEL database could hence be a valid tool for to support caterers, chefs, restaurants, nutritionists, municipalities, policy makers, to analyse different management options related to food and dietary planning.

### Database flexibility and directions for database improvement

The SEL database is not an exhaustive collection of CF and WF data for all the possible food items, although the bibliographic search covers a significant part of accessible data sources. The way the database is structured offers the possibility for further implementation actions:

1. Missing items can be included following the methodological framework proposed.
2. Items values and uncertainty can be implemented by adding new studies to level 1 and re-assessing uncertainty.
3. Level 1 data could be used for ex novo evaluations.
4. Technical and scientific users might like to make different assumption to further evaluate the uncertainty of specific items, in this case the clear and transparent description of the steps done to assign uncertainty labels allows to easily accept or reject any of the assumptions made in this paper and to implement the overall scheme making new attributions and decisions about the use of the items.
5. The role of geographic data distribution could be evaluated selecting and re-analysing level 1 data by regional groups, which might be relevant for indexes like the WF which are driven by climatic factors. The uncertainty analysis might allow to establish if CF and WF data attributed to an item are still sufficiently statistically robust to extract scientifically sound information. Regionalization of WF values might be relevant when combined with water scarcity studies for specific geographic areas of production.
6. The database flexibility allows to introduce new indexes or footprints by adding a level 1 collection of published data and including the relative levels 2, 3 and 4 calculated using the statistic recommendations provided in this paper. The same aggregation criteria proposed for the CF and WF might be used (groups, typologies, sub-typologies, items) for combined analyses of more indexes.

## Data Records

The SEL database is recorded in an Excel workbook called SUEATABLE_LIFE uploaded on Figshare^29^. It is composed of an introductory sheet to guide the users through the document plus 10 data sheets, 5 for the CF data analysis and 5 for the WF data analysis, and two additional sheets listing the typologies used for CF and WF data analysis and their description. The content of the 10 data sheets is here described. All the data analyses presented in this paper were done using the software STATA 14.2.

**SEL CF (or WF) for USERS** is a sheet that summarizes data in an easy to read form for expert and not expert users. It reports for each food item:

- the food group, the typology and the sub-typology corresponding to each item (Table 1)
- the item CF (or WF) value corresponding to the median CF (or WF) value reported for the same item in the SEL CF (or WF) ITEM STAT sheet.
- the level of uncertainty associated to the value of the item based on the three quality flags reported in the sheet SEL CF (or WF) ITEM STAT. The uncertainty associated to the ITEM footprint value is generically summarized as *high (H)* or *low (L)*.
- The suggestion for the optimal use of the data, namely if the items data is highly reliable or the uncertainty suggests to use a higher aggregation value.
- The alternative value of CF (or WF) that can be used for that specific item at higher level of aggregation (median of typology or sub-typology)

**CF (or WF) DATA SOURCES**: reports CF (or WF) values of food commodities collected from studies reported in the literature, screened using selection criteria and harmonized to report the same functional unit and system boundary. Information provided in this sheet for each input line are: name of group, typology, sub-typology, item (Table 1), CF or WF value after extraction from the publication and harmonization, type of source (journal, book, conference, EPD), full reference, publication year, system boundary at distribution, country of production, region of production, relevant notes, presence of the same value in other data collections, additional post farm gate emissions, transport (T), slaughtering (S), packaging (P) or conversion factor applied (cf).

**SEL CF (WF) ITEMS STAT**: reports for each food item the following statistical data: number of item values considered for the analysis (n), mean, median, standard deviation (SD), minimum (Min) and maximum (Max) observed values, Median Absolute Deviation (MAD), first quartile (Q1) and third quartile (Q3), coefficient of variation (CV), skewness (SK), kurtosis (KU) and P value of the Shapiro-Wilk normality test. Additionally, it reports the colour assigned to each quality flag (Flag 1 “size”, Flag 2 “outlier”, Flag 3 “adherence to normality”) and the suggested median value to be used on the basis of the scheme reported in Table 3. The statistical analysis for each item reported in this sheet is based on single data of CF (or WF) classified with that specific item entry name in the sheet SEL CF (or WF) DATA SOURCES.

**SEL CF (or WF) TYPOLOGIES:** reports for each food typology the following statistical data: number of item values (median) considered for the analysis (n), mean, median, standard deviation (SD), minimum (Min) and maximum (Max) observed values, Median Absolute Deviation (MAD), first quartile (Q1) and third quartile (Q3), coefficient of variation (CV), Interquartile Range (IQR), lower fence (LF) and upper fence (UF) for the estimate of outliers related to the specific typology. The statistical analysis for each typology reported in this sheet is based on median value of CF (or WF) of items classified under that specific typology and reported in the sheet SEL CF (or WF) ITEM STAT.

**SEL CF (or WF) sub-TYPOLOGIES:** reports for each food sub-typology the following statistical data: number of item values (median) considered for the analysis (n), mean, median, standard deviation (SD), minimum (Min) and maximum (Max) observed values, Median Absolute Deviation (MAD), first quartile (Q1) and third quartile (Q3), coefficient of variation (CV), Interquartile Range (IQR), lower fence (LF) and upper fence (UF), capital letters indicating significant difference among sub-typology medians within the same typology group based on the output of Kruskal-Wallis ANOVA test on ranks and pairwise multiple comparison procedures based on Dunn’s Method, significant differences between the sub-typology reported in the raw and the typology including the analysed sub-typology based on the Mann-Whitney Rank Sum test is also reported.

## Technical Validation

Given the high number of inputs and interconnected steps, each step was undertaken by at least two researchers. The literature review on LCA and WF studies followed the recommendations for collecting and synthesising relevant evidence reported in PRISMA^29^ (Preferred Reporting Items for Systematic Reviews and Meta-Analyses) and recommendations for CF data handling reported in Clune *et al*.^11^.

To classify typologies and sub-typologies that might be biologically and agronomical sound we had the specific support of biologists and agronomists.

The clarity of the “summary for users’ page” was tested with stakeholders of the food communication sector.

Concerning the choice of quality flags to attribute uncertainty to item data populations different options were evaluated and a group of scientific experts with different scientific background (statisticians, agronomists, biologist, ecologists) was involved for a final agreed choice.

To further support the correct choice of the attribution of uncertainty to each item based on the three flags, numerical data were supported by visual data of the distributions. Frequency distribution plots represented by adaptive kernel (Gaussian) density estimates and box and whisker plots were evaluated for each data population which did not provide immediate positive or negative evidence of reliability based on descriptive statistics and tests results.

**Fig. 4:**
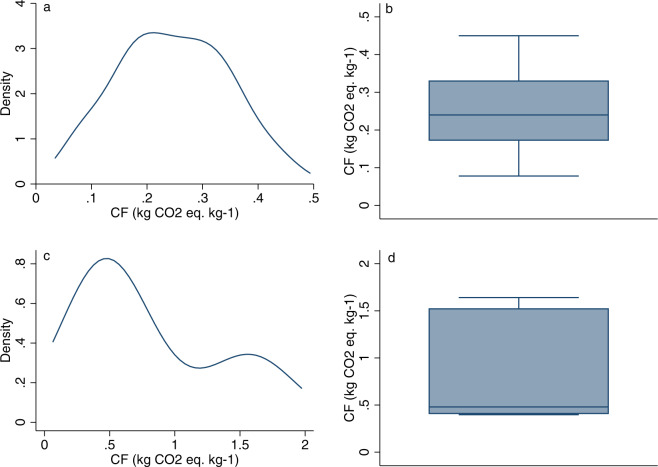
Kernel (Gaussian, bandwidth 0.0433) density estimate (**a**) and box-plot (**b**) of potato CF data. Kernel (Gaussian, bandwidth 0.3325) density estimate (**c**) and box-plot (**d**) of maize CF data. Empirical distribution for CF data of potato and maize as reported in studies listed in Level 1 of the database (data analysis done with STATA 14.2).

Two examples of such visualization and the consequent choices are reported in Fig. 4. Panels a and b (Fig. 4) show the empirical distribution of data population of CF values for the POTATO item, characterized by three green flags. Both kernel density and box-plot confirm the low level of dispersion of the empirical data range, the high level of concentration of the values around the centre of the distribution and the low density on both tails. The shape of the density function and the distance in the box-plot between Q1-Q3 and the median confirm the empirical adherence of this distribution to the normal distribution, as emerged by the SW test

Conversely, panels c and d (Fig. 4) shows the empirical distribution of the CF data population for the item MAIZE characterized by the following flags: “yellow” for size, “green” for outliers and “red” for adherence to the normal distribution, which indicated a medium level of uncertainty. The number of data in the population (n = 7) is sufficient to distinguish the mean value from the median value but is not optimal to compute and evaluate variability and shape of the distribution (Flag 3). The graphic visualization of the data in this case supports the attribution of the colour to the Flag3. The strong asymmetry of the data distribution and the high level of overall and central variability, highlighted by the range and the InterQuartile Range (IQR) of the box plot, confirm the median as the most suitable synthetic indicator of the data population and confirm the choice of the red flag resulting from the results of the Shapiro Wilk test.

## Usage Notes

Researchers and expert users could use any level of the database to extract information and could implement the database with additional data following the indications provided in this paper. Users that are not experts of data analysis can directly use the summary sheets ‘SEL CF (or WF) data for users’. The sheet provides CF or WF values for a list of items. The users also immediately find in the same raw the CF (or WF) value of typologies and sub-typologies corresponding to each item. Each item brings a quality indication which refers to the reliability of the data they have in the table. When the user finds the indication that an ITEM value has high uncertainty it means that speculations about this item value need caution and should be supported by additional data. For these items the use of the typology value might be more advisable. This could be the case for example of items represented by very few studies (1 or 2) or items represented by few studies (3 or 4) with output values very different from each other. If the users are looking for an item not included in the item list they can choose the median value of a typology or sub-typology of food commodity which they consider the closest to their missing object.

Users interested in using the methodological framework proposed or data present in the database are asked to cite this manuscript as well as the database and their relative DOI.

## Data Availability

No custom code has been used for generation or processing of the data or generation of figures.
